# Preadult Parental Diet Affects Offspring Development and Metabolism in *Drosophila melanogaster*

**DOI:** 10.1371/journal.pone.0059530

**Published:** 2013-03-26

**Authors:** Luciano M. Matzkin, Sarah Johnson, Christopher Paight, Therese A. Markow

**Affiliations:** 1 Department of Biological Sciences, University of Alabama in Huntsville, Huntsville, Alabama, United States of America; 2 Section of Cell and Developmental Biology, Division of Biological Sciences, University of California San Diego, La Jolla, California, United States of America; 3 Laboratorio Nacional de Genomica de la Biodiversidad, Centro de Investigaciones y Estudios Avancados, Irapuato, Guanajuato, Mexico; Queen Mary University of London, United Kingdom

## Abstract

When *Drosophila melanogaster* larvae are reared on isocaloric diets differing in their amounts of protein relative to sugar, emerging adults exhibit significantly different development times and metabolic pools of protein, glycogen and trigylcerides. In the current study, we show that the influence of larval diet experienced during just one generation extends into the next generation, even when that subsequent generation had been shifted to a standard diet during development. Offspring of flies that were reared on high protein relative to sugar underwent metamorphosis significantly faster, had higher reproductive outputs, and different metabolic pool contents compared to the offspring of adults from low protein relative to sugar diets. In addition, isofemale lines differed in the degree to which parental effects were observed, suggesting a genetic component to the observed transgenerational influences.

## Introduction

A wide spectrum of human health issues is known to be associated with prenatal and maternal factors. The ‘developmental origins of adult health and disease” hypothesis suggests that maternal nutrition, among other environmental factors, influences the risks for a range of adult health outcomes, such as obesity, cardiovascular disease, and the metabolic syndrome [1], [2]. In Warner and Ozanne’s [3] review of animal studies, a clear view emerges of how maternal diet may seriously impair fetal growth and the subsequent health of offspring even after they reach adulthood. Specific mechanisms of disruptions caused by various maternal nutritional deficiencies or excesses are under extensive investigation in vertebrate models [4]–[6].

*Drosophila* fruit flies afford a promising model for studies of human disease [7], [8], as considerable overlap exists in metabolic pathways and networks of humans and flies. The *Drosophila* model also can facilitate the investigation of pre-conception parental condition versus post-conception factors on subsequent offspring characteristics and performance. *Drosophila* reproduction is ovoviparous (development occurs outside the mother’s body) and the larval diet is easily manipulated. Ovoviparity thus provides an advantage for studies aimed at selectively examining the effect of parental condition at the time of conception apart from later-acting prenatal factors associated with pregnancy and lactation [9]–[11]. Studies already have revealed that differing levels of macro and micronutrients influence development and the metabolic phenotypes of emerging *Drosophila* adults and their offspring [1], [12]–[17].

Despite the marked increase in consumption of sweetened foods and beverages that has accompanied the obesity epidemic [18], the majority of experimental studies on the influence of prenatal diet on offspring health focus on protein deficiency and or excess dietary fat. Some exceptions, for vertebrate models, are Vickers et al [19] and references therein, where fructose has been of specific interest. In *Drosophila*, Matzkin et al [12] found that isocaloric larval diets that differed in their ratios of protein to sugar resulted in significant differences in the metabolic pools of protein, glycogen, and triglycerides of newly emerged adult flies. The diets in that study included those low in protein relative to sugar (LPS) as well as high in protein relative to sugar (HPS). In the LPS diet the ratio of sugar to protein was 9.8 compared to 2.6 for the HPS diet, and it produced emerging adults with significantly higher metabolic pools of triglycerides and glycogen relative to those reared on HPS.

Several observations led us to ask if the effects we observed might be carried over to the next generation and whether, if transgenerational effects are observed, they are genotype dependent. Two previous studies reported transgenerational diet effects in *Drosophila,* although these did not utilize isocaloric diets or examine genotype dependence. At the same time, striking effects of larval diet on adult metabolism have been found to exhibit significant genotype dependence [12], [13]. We thus were interested in the possible existence of transgenerational effects that also differ among genotypes. Specifically, we asked if these HPS and LPS larval developmental diets could influence phenotypes of the F1 progeny if those progeny all were reared on a standardized diet and whether genotype might modulate any observed parental effects. The phenotypes we measured were (1) egg production during the first days of adult life, (2) survival, developmental rates, and body mass, and (3) three metabolic pools (protein, glycogen and triglycerides) of the progeny. To address these questions, we reared individuals of five isofemale strains on the two diets, LPS and HPS, described above. All of the progeny of these flies, however, were then reared on a laboratory banana food, so that any observed differences could be attributed only to parental diet. Progeny from flies in the higher sugar diet were heavier (only in females), and experienced a longer metamorphosis (pupal) period. Furthermore, emerging adults differed significantly in their egg outputs and metabolic pools, depending upon parental diet.

## Materials and Methods

### Drosophila Isofemale Lines and Culture Conditions

As in our earlier study, we prepared diets that differed in their relative amounts of protein and sugar but were known to be isocaloric (110 calories/100 gm food) from assays performed by Exova Food Products Laboratory, Portland, Oregon USA [12]. HPS refers to the diet high in protein and low in sugar, while the low protein, high sugar diet is denoted by LPS. The relative protein:carbohydrate ratio of the HPS diet was determine to be 0.43 while that of the the LPS was 0.10 (Exova Food Products Laboratory, Portland, Oregon, USA) [12]. Each diet was composed of sucrose (VWR), active dry yeast (Genesee), yellow cornmeal (Genesee), and agar (Genesee). As in our previous study [12] HPS diet was prepared with 8 gm of sucrose and 32 gm of yeast, while the LPS used 32 gm of sucrose to 8 gm of yeast. Ingredients were mixed and boiled, followed by the addition of the antifungal methyl paraben (Genesee) dissolved in ethanol (Sigma-Aldrich), once the food had cooled to 55°C. Ten ml aliquots were then pipetted into 8-dram vials and allowed to cool until solid.

We utilized five isofemale lines of *D. melanogaster* collected from San Diego County in 2008. To rear the parental generation, we placed several hundred flies from each isofemale line in embryo chambers (Genesee Scientific) with 0.5% agar and a sprinkle of yeast to induce oviposition. Flies were allowed to oviposit for 24 hours after which we collected first instar larvae and placed them in 8-dram vials of the two diets described above (40 larvae/vial to avoid crowding effects). For each diet and isofemale line, 10 vials were set up.

Three days after eclosion the parental flies were placed in egg laying chambers with agar plates sprinkled with yeast. Parental generations were initiated to be certain that all eclosed at the same time. As in the earlier study, 40 first instar larvae per vial were set up but now were placed on a common standard-banana food (Markow and O’Grady 2005). Adults (F_1_) from each of the isofemale lines and parental diets (HPS or LPS) emerging from the standard banana food were then separated as virgins using CO_2_ anesthesia. We measured the effects of nutritionally distinct parental larval diet (HPS and LPS) on the (1) developmental time, (2) reproductive output in terms of number of eggs laid, (3) body size and (4) metabolic pools of the F_1_ progeny reared in a common diet.

### Developmental Time and Viability

As described above, first instar larvae (F_1_) were collected from egg collecting chambers and groups of 40 were gently transferred to vials of the standard-banana medium (10 vials per treatment). We examined vials daily and recorded when pupation was first observed, when adults first emerged, and the total number of flies produced.

### Reproductive Output

Newly emerged (less than 24 hours) female and male F_1_s were separated by sex and aged for three days in an un-yeasted standard-banana vial. Un-yeasted vials allow flies to undergo reproductive maturation without the confounding nutritional effects of live yeast. Females and males from the same line and parental diet were set up in pairs and allowed to mate once. After mating, males were removed and females individually were transferred to new un-yeasted standard-banana vial every 24 hours for four days. The number of eggs laid, as a proxy for reproductive output, in each vial was recorded daily.

### Parental Effects on Offspring Metabolic Pools

We asked if parental developmental diet influenced the size and metabolism of F_1s_ reared on a standard diet. As described above, we collected F_1_ first instar larvae from flies of each strain and parental diet and transferred them to standard-banana vials (40/vial). As flies emerged, adults (less than 24 hrs. old) were separated by sex, line and parental diet and frozen at −80°C. Once all adults were collected, flies were separated into groups of 5 based on sex, line and parental diet and dried in a 50°C oven for three days. The sample size for each isofemale line, sex, and parental treatment are given in Table S1. Dry mass was determined with a Cahn Model C-31 microbalance. Dried flies were homogenized in 1 ml of phosphate buffer (25 mmol/L KHPO_4_, pH 7.4) then centrifuged for two minutes at 13 Krpm. A total of 800 µl of supernatant was collected and frozen. Centrifugation of homogenates was performed to remove particulates that interfere with the colorimetric assays.

Colorimetric assays were performed for glycogen, triglycerides and total soluble protein using the same protocols as Matzkin et al. [12]. We measured absorbance for each metabolic pool using a Molecular Devices SpectraMax190 96-well microplate reader. Metabolic pools for a sample were measured in triplicate and the means of each triplicate were normalized by dry weight prior to analysis.

### Statistical Analysis

The total four-day egg production was analyzed using an ANOVA on square root transformed data. Developmental time was square root transformed and analyzed using a full factorial ANOVA with Parental Diet and Line as factors. Viability (number of individuals eclosed) was square root transformed prior to performing the ANOVA. Metabolic pool data were analyzed as a proportion of total dry mass, and thus these ratios were arcsine transformed prior to analysis (Sokal and Rohlf 1995). Total dry mass and the three metabolic pools were analyzed using a full factorial ANOVA with Parental Diet, Line and Sex as factors. For all statistical tests α was set at 0.05. All statistical analyses were performed using JMP 8.0 (SAS Institute Inc.).

## Results

### Developmental Time and Viability

Both egg to pupation and pupa to eclosion (metamorphosis) time are included in the egg to eclosion time, but only pupa to eclosion time showed any differences and was therefore analyzed further. Differences in the metamorphic period were observed at all levels of the analysis (Table 1). Flies whose parents developed on the HPS diet had a metamorphic stage that lasted four days, while flies whose parents were raised on the LPS diet had a significantly longer metamorphic stage (4.48±0.08 days). Finally, survival was not influenced by the parent’s larval diet, but rather by line and its interaction with diet (Table S2). Thus while survival was not affected by the larval diet of the parents, that portion of the development time spent in metamorphosis, was extended when sugar was high relative to protein.

**Table 1:** ANOVA of development time of the metamorphic state (pupa to first eclosion) of F_1_ progeny from isofemale lines of *D. melanogaster* that had been raised on larval diets HPS and LPS.

Source	*df*	SS	F Ratio
Parental Diet	1	0.134	29.2***
Line	4	0.098	5.2**
Parental Diet × Line	4	0.098	5.2**
Error	65	0.300	
Total	74	0.669	

* *P*<0.05,

** *P*<0.01,

*** *P*<0.001.

### Reproduction

**Figure 1:**
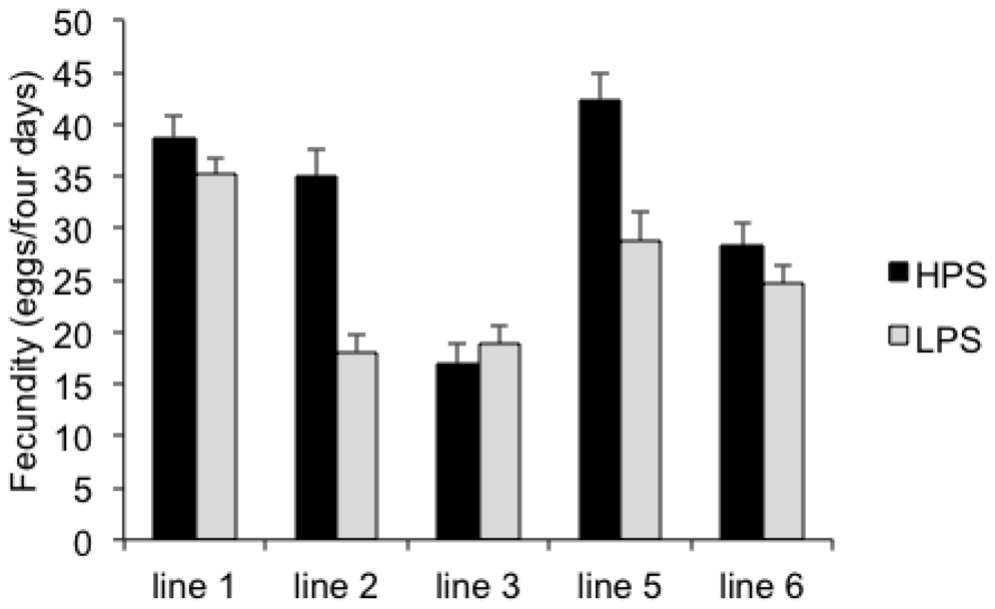
Isofemale line mean (± standard error) of eggs in four days b F_1_ females after a single mating.

The output of F_1_ flies, in terms of the number of eggs laid, from parents reared in the HPS diet was significantly greater than F_1_ flies from LPS parents (Figure 1, Table 2). Isofemale lines differed significantly, but with the exception of the F_1_ flies of line 3, in all cases we observed a greater egg output of flies whose parents were reared in the HPS diet (Figure 1). Those flies whose parents consumed less protein as larvae produced fewer eggs during the first four days of their adult life.

**Table 2:** ANOVA of mean number of eggs laid by F_1_ females in four days after a single mating.

Source	*df*	SS	F Ratio
Parental Diet	1	23.8	21.9***
Line	4	93.9	21.6***
Parental Diet × Line	4	22.0	5.1***
Error	184	200.0	
Total	193	339.4	

* *P*<0.05,

** *P*<0.01,

*** *P*<0.001.

### Dry Mass and Metabolic Pools

**Figure 2:**
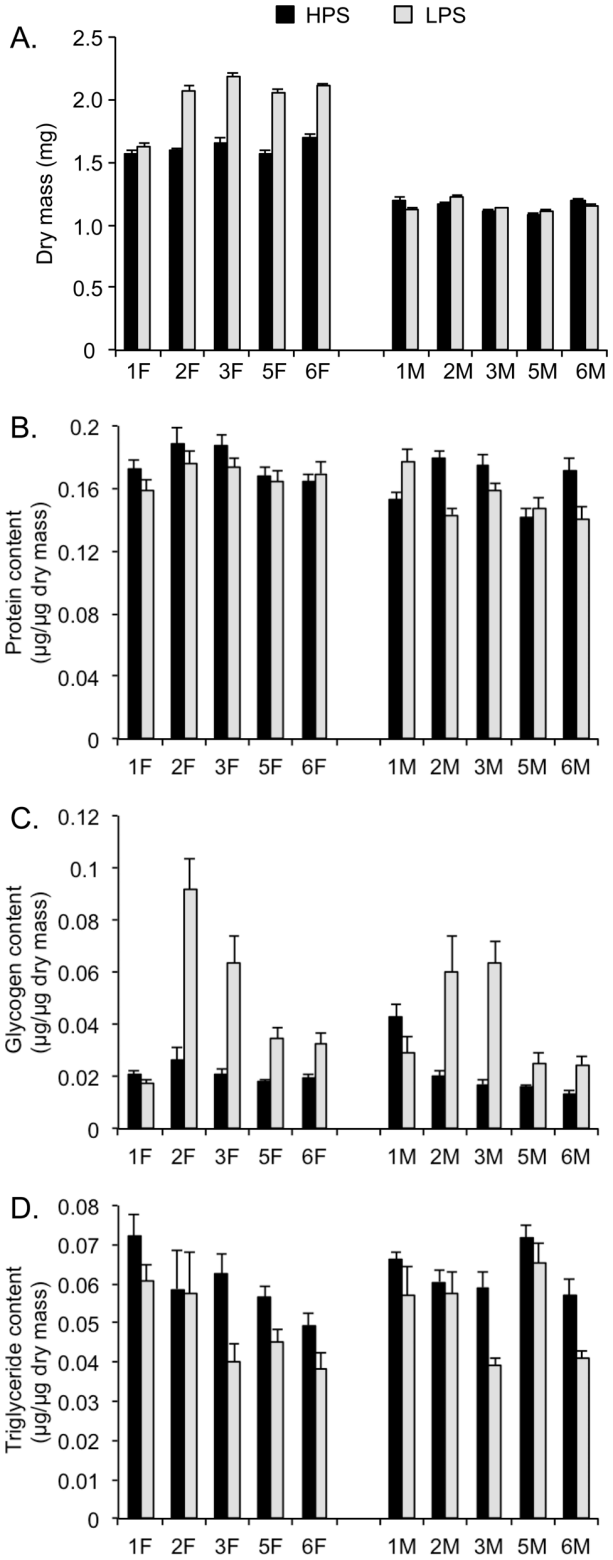
Dry mass (A), protein (B), glycogen (C), and triglycerides (D) content for F_1_ from isofemale lines of *D. melanogaster* raised on HPS or LPS diets. Values (means ± standard errors) are given for each isofemale line (numbers 1, 2, 3, 5 and 6) and sex, F = female and M = male.

Dry mass was most significantly affected by sex, which is not a surprise given that *D. melanogaster* females are normally larger than males (Figure 2a, Table 3). However, diet and line (as well as all higher order interactions terms) also were significant factors in the analysis (Figure 2a, Table 3). Owing to the large effect of sex on dry mass, we also performed the ANOVA for each sex separately; this was also done for each metabolic pool (see supplementary material). In the case of dry mass, significant parental diet effects only were observed in females (Table S3).

**Table 3:** ANOVA of dry mass for F_1_ from isofemale lines of *D. melanogaster* raised on larval diets HPS and LPS.

Source	*df*	SS	F Ratio
Parental Diet	1	1.96	287***
Line	4	0.762	28.0***
Sex	1	22.2	3262***
Parental Diet × Line	4	0.656	24.1***
Parental Diet × Sex	1	1.98	292***
Line × Sex	4	0.904	33.3***
Parental Diet × Line × Sex	4	0.283	10.4***
Error	190	1.29	
Total	209	31.6	

* *P*<0.05,

** *P*<0.01,

*** *P*<0.001.

The HPS diet significantly affected the protein level of the F_1_ (Table 4). After partitioning the analysis by sex, however, the effect was only present in females (Figure 2b, Table S4). In addition to diet and sex, we observed significant line effects, as well as several significant interaction terms (Table 4). For example, for line 1 the HPS diet produced higher protein levels in females while males had higher protein levels in the LPS parental diet treatment.

**Table 4:** ANOVA of protein content for F_1_ from isofemale lines of *D. melanogaster* raised on larval diets HPS and LPS.

Source	*df*	SS	F Ratio
Parental Diet	1	0.0076	9.02**
Line	4	0.0162	4.75**
Sex	1	0.0181	21.3***
Parental Diet × Line	4	0.0104	3.06*
Parental Diet × Sex	1	0.0003	0.33 ns
Line × Sex	4	0.0060	1.73 ns
Parental Diet × Line × Sex	4	0.0160	4.61**
Error	190	0.1617	
Total	209	0.2328	

* *P*<0.05,

** *P*<0.01,

*** *P*<0.001.

Glycogen content also was significantly influenced by parental diet, with the LPS diet producing significantly higher levels of glycogen (Table 5). Lines varied significantly in their glycogen levels. Four lines showed large increases in glycogen pools, but the degree of the increase differed among the lines (Figure 2c). Sex was not a significant factor, and similar results were observed when partitioning the analysis by sex (Table S5).

**Table 5:** ANOVA of glycogen content for F_1_ from isofemale lines of *D. melanogaster* raised on larval diets HPS and LPS.

Source	*df*	SS	F Ratio
Parental Diet	1	0.1651	84.3***
Line	4	0.1172	15.0***
Sex	1	0.0034	1.73 ns
Parental Diet × Line	4	0.1551	19.8***
Parental Diet × Sex	1	0.0032	1.62 ns
Line × Sex	4	0.0500	6.38***
Parental Diet × Line × Sex	4	0.0080	1.01 ns
Error	190	0.3722	
Total	209	0.8914	

* *P*<0.05,

** *P*<0.01,

*** *P*<0.001.

Triglyceride content (Figure 2d), on the other hand, was significantly elevated in both sexes when parents had developed in the HPS diet (Table 6). In addition to the significant diet effect, lines also differed, as some lines had higher triglyceride content than others (Table 6). Sex did not affect triglyceride levels although the Line × Sex interaction was significant. The sex-specific analysis yielded line effects. (Table S6).

**Table 6:** ANOVA of triglyceride content for F_1_ from isofemale lines of *D. melanogaster* raised on larval diets HPS and LPS.

Source	*df*	SS	F Ratio
Parental Diet	1	0.0350	29.3***
Line	4	0.0425	8.91***
Sex	1	0.0046	3.82
Parental Diet × Line	4	0.0114	2.39
Parental Diet × Sex	1	0.0001	0.05
Line × Sex	4	0.0141	2.96*
Parental Diet × Line × Sex	4	0.0007	0.15
Error	190	0.2264	
Total	209	0.3497	

* *P*<0.05,

** *P*<0.01,

*** *P*<0.001.

## Discussion

Larval protein to sugar ratios significantly impact not only adult characters [12], but those of their offspring as well (this study), even when those offspring themselves are reared on an identical diets. The standardized banana diet failed to eliminate the effects of parental rearing condition on the next generation. While survival was unaffected by parental diet, the LPS parental dietreduced the number of eggs the offspring produced during the four days of adult life. Because the life expectancy of adult *D. melanogaster* in nature has been estimated at less than a week [20], days four to seven of adult life (our reproductive observation period) should correspond to reproductive fitness in the wild. Clearly F_1_ egg output was compromised by parental developmental diet. Differences also existed in female body size: female offspring of LPS parents were much larger, although it did not result in any reproductive advantage as predicted by life history theory [17].

Even more striking were the parental effects on the metabolic pools of their offspring. In two cases, protein and glycogen, parental metabolic pools predicted the metabolic pools of their offspring (Figure S1). Parents reared on the LPS diet had lower metabolic pools of protein compared to those reared on high protein and the same was true of their offspring. The LPS diet produced parents that had high levels of glycogen compared to the HPS parents and their offspring differed from each other in the same direction. Triglyceride pools also were affected by parent diet, but in the opposite direction from the parental pools (Figure S1). Rather than being higher in the LPS, as in the parents, they were significantly lower. Importantly, isofemale lines varied in their responses in all the metabolic pools, indicating significant genetic variation in the way individuals respond to the diets of their parents. Not all genotypes respond identically, a situation observed in the parental generation [12], [13] as well as in the offspring. These genotype × environment interactions have significant implications for human health, as some individuals, families and/or populations may be more vulnerable than others to the influence of parental nutrition than others. Differences among isofemale strains can be exploited to examine the basis for individual vulnerability to environmentally induced metabolic disorders.

Our experiments were not designed to separate maternal versus paternal contributions to the observed trans-generational effect [21]. Examining the relative roles of maternal and paternal diets will be a large undertaking and is planned as a future study. It is tempting to conclude that the effects observed are attributable primarily to maternal rearing diet, as *Drosophila* eggs are large gametes that support embryonic development. Adult sexual maturity, however, especially gametogenesis, could have been delayed in both sexes of the progeny of LPS parents, contributing to the lower egg output observed. Ng et al [22] recently showed that in mice, paternal diet could significantly influence the metabolism of their daughters. Without additional experiments maternal and paternal contributions to the observed effects cannot yet be disentangled. In *D. melanogaster* as well as in another dipteran, *Thelostylinus angusticollis,* neither of which show paternal investment in offspring, the influence of paternal diet quality offspring phenotype was clearly shown [21], [23], indicating that paternal effects certainly may contribute to our observations.

Also curious is that while the metabolic pools of protein and glycogen were high in the LPS parents and their offspring, this was not true of the triglycerides. In the present study, all offspring were reared on the identical standard laboratory *Drosophila* diet of banana medium, which differed from both of the parental diets in that it was lower in fat, protein, carbohydrates and total calories per unit volume. Yet the responses of progeny from different parental diets differed significantly from each other. Reduction in the triglycerides may reflect some interaction between parental and offspring diets that deserves attention in a future study. The degree to which offspring diet can correct for negative metabolic effects of parental condition or, alternatively, exacerbate them, remains obscure. Likewise, the mechanism(s) underlying the observed trans-generational effects remain unknown, but could reflect a range of processes. For example, rats having developed in a protein-reduced prenatal environment were reported to exhibit feeding behaviors that in turn influenced their body compositions [24]. A similar possibility cannot be excluded in the case of *Drosophila*. In vertebrates, a number of studies have now shown that prenatal nutrition influences metabolic expression profiles in later life [25]–[30] as well as epigenetic modifications in rodents and humans [19], [31]–[33]. High sugar as well as low or high protein could be responsible. There could also be some effect of micronutrient differences associated with the use of yeast as the protein source. For example, obesity in rodents can be a function of prenatal exposure to low protein diets [27], as well as to high protein diets [34], suggesting that different developmental disturbances of nutrient balance may induce common metabolic responses.

*Drosophila* offer a relatively inexpensive high-throughput system for studies of parental effects of diet on a wide range of offspring traits. Because there is no internal development, the *Drosophila* system allows us to eliminate gestational effects and directly target the role of parental condition or larval nutrition or insults. In addition, larval diets can effectively be pulsed and switched during particular developmental stages. Despite the conformity in offspring diets, we found that parental nutrition exerts a significant effect on the next generation. Future studies will determine if these trans-generational effects last into future generations as well as help to understand their bases. For example, the relative paternal and maternal contributions need to be separated. Additionally, the specific mechanisms remain unknown. The role of diet-induced changes in gene regulation through epigenetic or metabolic factors must be determined. While the metabolic profiles of offspring clearly are influenced by parental rearing diet, it remains unclear whether these changes can modify additional offspring traits such as longevity or resistance to stress or disease. Our laboratories currently are investigating these questions.

## Supporting Information

Figure S1 Isofemale line mean (± standard error) four-day fecundity for the parents (P) raised on the HPS or LPS diets and their offspring (F_1_) who were raised in a common standard banana diet.
(DOCX)

Table S1 Samples size for all measurements. **A.** Sample size of females for egg laying data. **B.** Sample size of number of vials of 40 larvae for development time and survival data. **C.** Number of homogenates of 5 flies used for dry mass and metabolic pools analysis
(DOCX)

Table S2 ANOVA of viability of F_1_ from isofemale lines of *D. melanogaster* raised on larval diets HPC and LPC.
(DOCX)

Table S3 ANOVA of dry mass for F_1_ from isofemale lines of *D. melanogaster* raised on larval diets HPC and LPC.
(DOCX)

Table S4 ANOVA of protein content for F_1_ from isofemale lines of *D. melanogaster* raised on larval diets HPC and LPC.
(DOCX)

Table S5 ANOVA of glycogen content for F_1_ from isofemale lines of *D. melanogaster* raised on larval diets HPC and LPC.
(DOCX)

Table S6 ANOVA of triglyceride content for F_1_ from isofemale lines of *D. melanogaster* raised on larval diets HPC and LPC.
(DOCX)
