# Supplementary material for: Preadult Parental Diet Affects Offspring Development and Metabolism in Drosophila melanogaster
Source: PLoS One. 2013 Mar 26;8(3):e59530. doi: 10.1371/journal.pone.0059530 (PMC3608729; doi:10.1371/journal.pone.0059530)
Supplement: Figure S1 — Isofemale line mean (± standard error) four-day fecundity for the parents (P) raised on the HPS or LPS diets and their offspring (F1) who were raised in a common standard banana diet. (DOCX) [file pone.0059530.s001.docx]

**Figure S1.** Isofemale line mean (± standard error) four-day fecundity for the parents (P) raised on the HPS or LPS diets and their offspring (F_1_) who were raised in a common standard banana diet.
