# Supplementary material for: Preadult Parental Diet Affects Offspring Development and Metabolism in Drosophila melanogaster
Source: PLoS One. 2013 Mar 26;8(3):e59530. doi: 10.1371/journal.pone.0059530 (PMC3608729; doi:10.1371/journal.pone.0059530)
Supplement: Table S1 — Samples size for all measurements. A. Sample size of females for egg laying data. B. Sample size of number of vials of 40 larvae for development time and survival data. C. Number of homogenates of 5 flies used for dry mass and metabolic pools analysis (DOCX) [file pone.0059530.s002.docx]

**TABLE S1:** Samples size for all measurements.

**A.** Sample size of females for egg laying data

| **Diet** | **Line** | ***F1*** | ***Parent*** |
| --- | --- | --- | --- |
| LPS | 1 | 20 | 18 |
| LPS | 2 | 18 | 18 |
| LPS | 3 | 20 | 17 |
| LPS | 5 | 19 | 18 |
| LPS | 6 | 19 | 19 |
| HPS | 1 | 20 | 19 |
| HPS | 2 | 20 | 18 |
| HPS | 3 | 19 | 20 |
| HPS | 5 | 20 | 20 |
| HPS | 6 | 19 | 20 |

**B.** Sample size of number of vials of 40 larvae for development time and survival data

| **Diet** | **Line** | ***F1*** |
| --- | --- | --- |
| LPS | 1 | 8 |
| LPS | 2 | 8 |
| LPS | 3 | 8 |
| LPS | 5 | 8 |
| LPS | 6 | 8 |
| HPS | 1 | 8 |
| HPS | 2 | 8 |
| HPS | 3 | 8 |
| HPS | 5 | 8 |
| HPS | 6 | 8 |

**C.** Number of homogenates of 5 flies used for dry mass and metabolic pools analysis

| **Diet** | **Line** | ***F1*** | ***n*** |
| --- | --- | --- | --- |
| HPS | 1 | Female | 14 |
| HPS | 1 | Male | 15 |
| HPS | 2 | Female | 9 |
| HPS | 2 | Male | 10 |
| HPS | 3 | Female | 10 |
| HPS | 3 | Male | 10 |
| HPS | 5 | Female | 10 |
| HPS | 5 | Male | 10 |
| HPS | 6 | Female | 9 |
| HPS | 6 | Male | 9 |
| LPS | 1 | Female | 10 |
| LPS | 1 | Male | 10 |
| LPS | 2 | Female | 9 |
| LPS | 2 | Male | 8 |
| LPS | 3 | Female | 15 |
| LPS | 3 | Male | 15 |
| LPS | 5 | Female | 10 |
| LPS | 5 | Male | 8 |
| LPS | 6 | Female | 9 |
| LPS | 6 | Male | 10 |
