# Supplementary material for: Preadult Parental Diet Affects Offspring Development and Metabolism in Drosophila melanogaster
Source: PLoS One. 2013 Mar 26;8(3):e59530. doi: 10.1371/journal.pone.0059530 (PMC3608729; doi:10.1371/journal.pone.0059530)
Supplement: Table S2 — ANOVA of viability of F1 from isofemale lines of D. melanogaster raised on larval diets HPC and LPC. (DOCX) [file pone.0059530.s003.docx]

**TABLE S2** ANOVA of viability of F_1_ from isofemale lines of *D. melanogaster* raised on larval diets HPC and LPC.

| **Source** | ***df*** | **SS** | **F Ratio** |
| --- | --- | --- | --- |
| Parental Diet | 1 | 0.028 | 0.63 |
| Line | 4 | 0.577 | 3.22 * |
| Parental Diet × Line | 4 | 1.348 | 7.54 *** |
| Error | 65 | 2.907 |  |
| Total | 74 | 4.873 |  |

* *P* < 0.05, ** *P* < 0.01, *** *P* < 0.001
