# Supplementary material for: Preadult Parental Diet Affects Offspring Development and Metabolism in Drosophila melanogaster
Source: PLoS One. 2013 Mar 26;8(3):e59530. doi: 10.1371/journal.pone.0059530 (PMC3608729; doi:10.1371/journal.pone.0059530)
Supplement: Table S3 — ANOVA of dry mass for F1 from isofemale lines of D. melanogaster raised on larval diets HPC and LPC. (DOCX) [file pone.0059530.s004.docx]

**TABLE S3** ANOVA of dry mass for F_1_ from isofemale lines of *D. melanogaster* raised on larval diets HPC and LPC.

|  | **Source** | ***df*** | **SS** | **F Ratio** |
| --- | --- | --- | --- | --- |
| Females | Parental Diet | 1 | 3.968 | 367.1 *** |
|  | Line | 4 | 1.532 | 35.5 *** |
|  | Parental Diet × Line | 4 | 0.875 | 20.3 *** |
|  | Error | 95 | 1.024 |  |
|  | Total | 104 | 7.720 |  |
|  |  |  |  |  |
| Males | Parental Diet | 1 | 5.6×10^-5^ | 0.02 ns |
|  | Line | 4 | 0.113 | 10.0 *** |
|  | Parental Diet × Line | 4 | 0.060 | 5.2 *** |
|  | Error | 95 | 0.268 |  |
|  | Total | 104 | 0.443 |  |

* *P* < 0.05, ** *P* < 0.01, *** *P* < 0.001
