# Supplementary material for: Preadult Parental Diet Affects Offspring Development and Metabolism in Drosophila melanogaster
Source: PLoS One. 2013 Mar 26;8(3):e59530. doi: 10.1371/journal.pone.0059530 (PMC3608729; doi:10.1371/journal.pone.0059530)
Supplement: Table S4 — ANOVA of protein content for F1 from isofemale lines of D. melanogaster raised on larval diets HPC and LPC. (DOCX) [file pone.0059530.s005.docx]

**TABLE S4** ANOVA of protein content for F_1_ from isofemale lines of *D. melanogaster* raised on larval diets HPC and LPC.

|  | **Source** | ***df*** | **SS** | **F Ratio** |
| --- | --- | --- | --- | --- |
| Females | Parental Diet | 1 | 0.0025 | 2.8 ns |
|  | Line | 4 | 0.0098 | 2.7 * |
|  | Parental Diet × Line | 4 | 0.0021 | 0.6 ns |
|  | Error | 95 | 0.0853 |  |
|  | Total | 104 | 0.0988 |  |
|  |  |  |  |  |
| Males | Parental Diet | 1 | 0.0054 | 6.7 * |
|  | Line | 4 | 0.0119 | 3.7 ** |
|  | Parental Diet × Line | 4 | 0.0241 | 7.5 *** |
|  | Error | 95 | 0.0765 |  |
|  | Total | 104 | 0.1157 |  |

* *P* < 0.05, ** *P* < 0.01, *** *P* < 0.001
