# Supplementary material for: Preadult Parental Diet Affects Offspring Development and Metabolism in Drosophila melanogaster
Source: PLoS One. 2013 Mar 26;8(3):e59530. doi: 10.1371/journal.pone.0059530 (PMC3608729; doi:10.1371/journal.pone.0059530)
Supplement: Table S6 — ANOVA of triglyceride content for F1 from isofemale lines of D. melanogaster raised on larval diets HPC and LPC. (DOCX) [file pone.0059530.s007.docx]

**TABLE S6** ANOVA of triglyceride content for F_1_ from isofemale lines of *D. melanogaster* raised on larval diets HPC and LPC.

|  | **Source** | ***df*** | **SS** | **F Ratio** |
| --- | --- | --- | --- | --- |
| Females | Parental Diet | 1 | 0.0191 | 11.3 *** |
|  | Line | 4 | 0.0293 | 4.3 ** |
|  | Parental Diet × Line | 4 | 0.0069 | 1.03 ns |
|  | Error | 95 | 0.1601 |  |
|  | Total | 104 | 0.2233 |  |
|  |  |  |  |  |
| Males | Parental Diet | 1 | 0.0159 | 22.8 *** |
|  | Line | 4 | 0.0263 | 9.4 *** |
|  | Parental Diet × Line | 4 | 0.0051 | 1.82 ns |
|  | Error | 95 | 0.0663 |  |
|  | Total | 104 | 0.1231 |  |

* *P* < 0.05, ** *P* < 0.01, *** *P* < 0.001
